# Pregnancy After Beating Thyroid Cancer: A Case Series

**DOI:** 10.7759/cureus.72122

**Published:** 2024-10-22

**Authors:** Shruti Ganti, Shanthi Ethirajan, Jayashree Srinivasan, Tharaka Senathirajah

**Affiliations:** 1 Department of Obstetrics and Gynaecology, Saveetha Medical College and Hospitals, Saveetha Institute of Medical and Technical Sciences, Saveetha University, Chennai, IND

**Keywords:** beta-hcg, maternofetal outcome, pregnancy, thyroid cancer, thyroid-stimulating hormone (tsh)

## Abstract

Thyroid cancer is common among women of reproductive age and is a frequently occurring cancer during pregnancy, following breast cancer. Contributing factors include radiation exposure, iodine deficiency, and genetic conditions like multiple endocrine neoplasia type 2 (MEN2). Pregnancy significantly impacts thyroid function, leading to gland enlargement and altered thyroid stimulating hormone (TSH) levels due to hormonal changes, including elevated human chorionic gonadotropin (hCG) and estrogen. When differentiated thyroid cancer (DTC), particularly papillary thyroid carcinoma (PTC), is diagnosed during pregnancy or shortly after, careful management is essential. Treatment usually involves total thyroidectomy, with radioactive iodine therapy delayed until after childbirth. A review of cases indicates that women with PTC can have successful pregnancies with close medical supervision. Despite the complexities of cancer treatment during pregnancy, outcomes for both mother and baby are generally positive. These cases emphasize the importance of a collaborative approach in managing thyroid cancer during pregnancy and highlight the need for further research to optimize treatment and outcomes for both mother and child.

## Introduction

Thyroid cancer is more prevalent in females of reproductive age and ranks as the second most common cancer during pregnancy, following breast cancer, with an incidence of 14 per 100,000 live births [1]. Risk factors include exposure to ionizing radiation, iodine deficiency, and the genetic syndrome multiple endocrine neoplasia type 2 (MEN2), which affects about 1-2% of thyroid cancer patients [2]. Sex hormones also play a role, as differentiated thyroid cancer (including papillary and follicular types) is more common in women of childbearing age. During pregnancy, the thyroid gland enlarges by about 30%, and thyroid-stimulating hormone (TSH) levels fluctuate, decreasing in the first trimester before normalizing. Until the 12th week of gestation, maternal thyroxine is the primary source of thyroid hormones for the fetus, as the fetal thyroid cannot concentrate iodine [3]. Monitoring thyroid function with gestational age-specific reference ranges is essential, especially for thyroid cancer patients, as TSH levels guide adjustments in suppressive levothyroxine therapy [4].

Pregnancy affects the thyroid gland through two main mechanisms: increased levels of human chorionic gonadotropin (hCG) and elevated estrogen levels. hCG stimulates TSH receptors, increasing thyroid activity and lowering TSH levels, particularly in the first 12 weeks [5]. This effect is also seen in conditions like gestational trophoblastic diseases, where elevated hCG can cause hyperthyroidism in a small percentage of patients [6].

Thyroid nodules, common during pregnancy, can increase in size or number. Differentiated thyroid cancer (DTC) diagnosed during pregnancy or within 12 months postpartum requires careful evaluation, as DTC is the second most common cancer diagnosed during pregnancy and postpartum. If a thyroid nodule is detected, malignancy should be considered, and fine needle aspiration cytology may be needed to distinguish benign from malignant nodules [7]. Total or near-total thyroidectomy is the standard treatment for thyroid cancer, but surgery during pregnancy must balance maternal and fetal outcomes. There is no evidence that thyroid cancer diagnosis necessitates pregnancy termination. The Endocrine Society recommends delaying thyroidectomy until after delivery for non-advanced cases or performing it in the second trimester for those with more aggressive disease [8]. Radioactive iodine therapy is postponed until after delivery and breastfeeding cessation.

Radioactive iodine therapy is contraindicated during pregnancy due to risks of fetal hypothyroidism and cognitive impairment. If necessary post-delivery, breastfeeding should stop at least six weeks before treatment. Although there is no evidence that radioactive iodine affects fertility or future pregnancies, it is advised to avoid conception for 12 months post-treatment. Levothyroxine (LT4) therapy is required during pregnancy for patients awaiting surgery or post-thyroidectomy, and monthly monitoring of thyroid function is essential.

Papillary thyroid carcinoma (PTC) incidence has risen in recent years [9-12]. Managing thyroid cancer during pregnancy presents challenges regarding the timing of surgical and medical interventions. In PTC patients, careful management of hyperthyroidism and hypothyroidism is crucial to prevent fetal complications and ensure normal brain development. Propylthiouracil is preferred in early pregnancy due to the teratogenic risks associated with methimazole, while hypothyroidism management is essential to reducing miscarriage risk and supporting fetal brain development [7-8]. Additionally, the potential effects of radioactive iodine therapy on reproductive health must be considered in these patients.

## Case presentation

Case 1

 A 32-year-old female, para 2, live 2, abortion 1 (P2L2A1), was diagnosed with papillary thyroid carcinoma (PTC) in 2018. She had a history of neck swelling from 2013 to 2018, which increased in size during and after her first pregnancy in 2016. The swelling was gradual and progressive. There was no history of dysphagia, stridor, dyspnea, voice changes, vomiting, loss of appetite, weight loss, or edema. She also reported irregular menstrual cycles over the past two years, occurring every 2-3 months for 3-4 days. There was no significant family history.

On examination, bilateral thyroid nodules were noted in the inferior poles and the left superior pole. Papillary carcinoma was confirmed through ultrasound-guided fine needle aspiration cytology (FNAC), which showed Bethesda category 5 in the superior pole, indicating suspicion of papillary carcinoma. A total thyroidectomy with lymph node dissection was performed in 2018. Histopathological examination revealed classic papillary thyroid carcinoma, pT2pNxpM0, measuring 3 cm, with lymphovascular space invasion and confinement to the thyroid gland, with clear margins.

**Figure 1:**
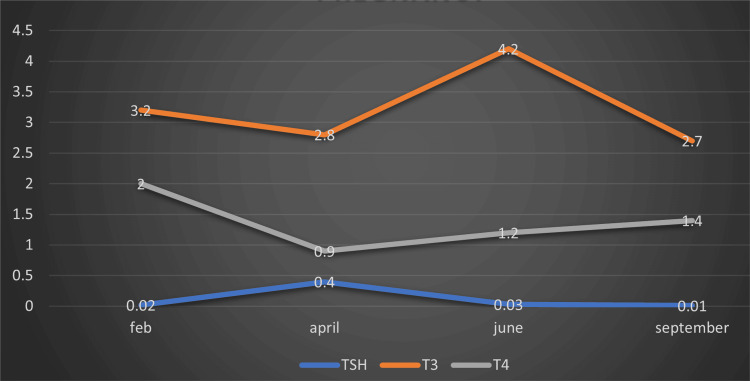
Thyroid function tests during the period of pregnancy in Case 1 T3: triiodothyronine (normal: 2.77-5.27 pg/mL); T4: thyroxine (normal: 0.78-2.19pgl/L); TSH: thyroid stimulating hormone (normal: 0.46–4.68 mIU/L).

At the time of diagnosis, the patient was in her second pregnancy, and an ultrasound showed a single live intrauterine gestation (SLIUG) at 6 weeks and 6 days. The pregnancy was terminated via medical termination (MTP) due to the need for radioactive iodine therapy. Post-operatively, she was started on 175 mcg of thyroxine daily. In 2020, two years after thyroidectomy, the patient spontaneously conceived again, confirmed by a urine pregnancy test at 45 days of amenorrhea. She was booked and immunized at Saveetha Medical College, India. Her pregnancy was uneventful, with thyroid hormone levels remaining within normal limits throughout. She was maintained on 200 mcg of thyroxine during pregnancy, and her TSH levels remained stable (as shown in Figure 1). Continuous suppressive therapy helped control her thyroid status.

The patient delivered a healthy female baby weighing 3 kg at 38 weeks and 6 days via normal vaginal delivery. The Apgar scores were 8/10 and 9/10, with normal TSH levels in the baby. Post-natal follow-up was uneventful, and the baby had no thyroid disorders, abnormal otoacoustic emissions (OAE), congenital anomalies, or genetic syndromes. Pediatric follow-ups were normal. The patient's serum thyroglobulin levels remained stable, with sustained TSH suppression (0.01-0.71 mIU/L) before, during, and after pregnancy, maintained with oral levothyroxine. The patient felt well throughout her pregnancy and post-natal period, with no significant fluctuations in thyroid function.

Case 2

 A 28-year-old female, para 1, living 0 (P1L0), was diagnosed with papillary thyroid cancer (PTC) in 2011 after presenting with insidious neck swelling, hoarseness of voice, and dysphagia without loss of appetite, weight loss, or significant family history. Examination revealed uniform swelling of the thyroid gland. She underwent total thyroidectomy with right neck nodal dissection, followed by postoperative radioactive iodine therapy. The final histopathology report confirmed papillary carcinoma of the thyroid with metastasis.

**Figure 2:**
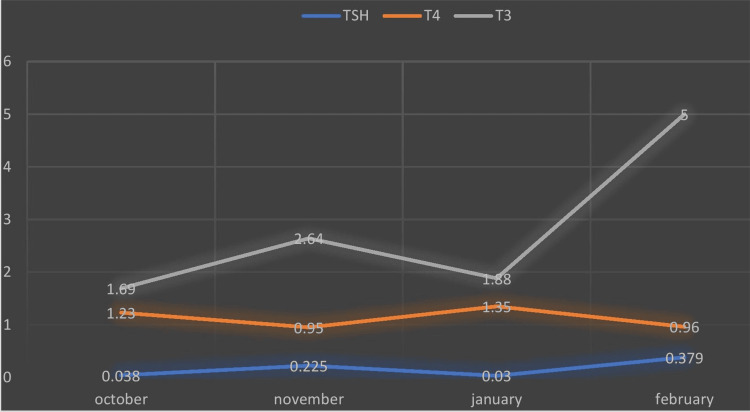
Thyroid function tests in Case 2 TSH: Thyroid stimulating hormone; T3: Tri iodothyronine; T4: Thyroxine.

Her first pregnancy occurred 10 years after her cancer treatment, through spontaneous conception, with thyroid levels controlled via thyroxine supplementation. All antenatal scans were normal. However, at 34 weeks, she experienced an intrauterine death of unknown cause, with absent fetal movements but no fever, bleeding, or abdominal pain. She delivered via lower segment cesarean section (LSCS) after a failed labor induction. Her second pregnancy, in February 2023, was also spontaneously conceived. Antenatal scans revealed no anomalies, and her thyroid function tests during all trimesters were within normal limits. Thyroxine dose adjustments were made based on TSH levels, with the final dose being 162.5 mcg. TSH and T4 levels during pregnancy are shown in Figure 2.

In her current pregnancy, she underwent elective LSCS at 38 weeks and delivered a healthy boy weighing 2.56 kg with APGAR scores of 8/10 and 9/10. Her postoperative period was uneventful, with no significant fluctuations in her postnatal thyroid function tests. The baby showed no signs of thyroid disorders, abnormal otoacoustic emissions (OAE), congenital anomalies, or genetic syndromes. Pediatric follow-ups for the baby were normal.

Case 3

A 32-year-old female, para 1, living 1 (P1L1), was diagnosed with papillary thyroid cancer (PTC) in 2021. She had a history of gradual neck swelling for eight years, which increased in size and was associated with dyspnea, pricking pain, and irregular menstrual cycles. On examination, a 6x7 cm swelling was noted in front of her neck. Ultrasound of the neck revealed a colloid nodule in the right thyroid lobe, and fine needle aspiration cytology (FNAC) indicated colloid goitre with cystic changes (Bethesda Category 2). A CT scan showed multinodular goitre with cystic degeneration, likely neoplastic with lymph node invasion. She underwent a total thyroidectomy with neck dissection in 2021. The final histopathology report confirmed papillary carcinoma of the thyroid, classic type (PT3a, N0).

**Figure 3:**
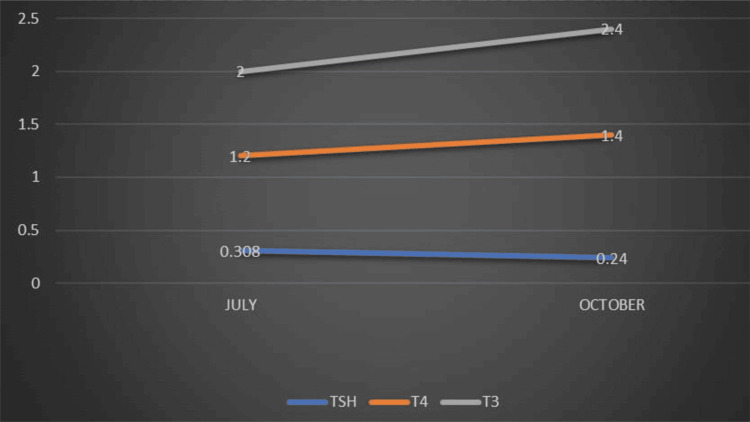
Thyroid function tests in Case 3 TSH: Thyroid stimulating hormone; T3: Tri iodothyronine; T4: Thyroxine.

She conceived her second pregnancy spontaneously in 2023. Her first pregnancy was 13 years earlier, during which her thyroid function was normal. She delivered a 2.7 kg male baby via lower segment cesarean section (LSCS) due to an arrest of descent in labor. After the birth, she developed neck swelling, leading to the diagnosis and treatment of papillary thyroid cancer, followed by thyroid hormone replacement with 100 mcg thyroxine. The TSH levels during her second pregnancy are shown in Figure 3.

During her second pregnancy in 2023, an ultrasound at 37 weeks revealed a growth discrepancy, with the fetus corresponding to 34 weeks gestation, and the patient was diagnosed with intrauterine growth restriction (IUGR) with doppler changes. She underwent an emergency LSCS, delivering a healthy baby girl weighing 1.9 kg, small for gestational age (SGA), with APGAR scores of 8/10 and 9/10. The baby was admitted to the neonatal intensive care unit (NICU) due to low birth weight but had no congenital defects. Otoacoustic emission (OAE) and thyroid stimulating hormone (TSH) levels were normal. There were no fluctuations in the patient's postnatal thyroid function tests, and pediatric follow-ups were normal.

## Discussion

The provided scenarios emphasize many crucial clinical aspects. The delay in diagnosis of thyroid cancer may occur during pregnancy because the symptoms can be mistaken for other pregnancy-related symptoms. Additionally, radioactive iodine therapy should not be used during pregnancy. However, choosing to delay the treatment may result in increased worries and require careful monitoring after giving birth. Furthermore, individuals who previously had thyroid cancer may encounter diverse obstetric outcomes, such as higher chances of intrauterine growth restriction (IUGR) and fetal mortality within the uterus. This necessitates customized monitoring during pregnancy and careful planning for delivery.

It is essential to closely monitor patients who have thyroid cancer and become pregnant in order to track the progression of the disease and offer the best possible care during pregnancy. Thyroid cancer identified during pregnancy might lead to concerns regarding the timing of therapy and the possibility of health issues for both the mother and the newborn [13]. Nevertheless, young adults diagnosed with differentiated thyroid cancer typically have a favorable prognosis, and pregnant women with thyroid cancer experience similar results to non pregnant women with comparable illness. It is crucial to regularly evaluate and modify thyroid hormone levels during pregnancy after being diagnosed with thyroid cancer in order to ensure the well-being of both the mother and the fetus [14].

Untreated thyroid cancer during pregnancy can lead to the reappearance and advancement of structural illness, negative outcomes in obstetrics, improper growth of the child, and the development of thyroid nodules. Monitoring and managing thyroid cancer in pregnant women is essential to minimize these possible hazards [15]. Maternal thyroid cancer is unlikely to have significant negative consequences on pregnancy outcomes, except for abnormal weight gain during pregnancy. There was no notable disparity in thyroid stimulating hormone (TSH) levels in neonates born to moms with thyroid cancer compared to those delivered to mothers without thyroid cancer. Additional research is required to examine the effects of prolonged thyroid function on cognitive function in the children of women diagnosed with thyroid cancer. The study emphasized the significance of maintaining thyroid hormone levels within the appropriate range during pregnancy to ensure normal fetal growth [16].

Pregnancies complicated by thyroid cancer exhibit elevated rates of venous thromboembolism (VTE) and necessitate transfusions while maintaining similar overall outcomes for newborns. Thyroid cancer identified during pregnancy does not have a substantial effect on the health of the mother and baby, and pregnant women with thyroid cancer tend to have positive outcomes regardless of when the disease is diagnosed [17,18]. Maternal thyroid function test abnormalities, including subclinical hypothyroidism, isolated hypothyroxinemia, and thyroid peroxidase antibody positive, have been shown to increase the likelihood of premature birth. Nevertheless, the study did not discover a clear connection between the stage of maternal thyroid cancer and the rates of preterm birth [19,20].

In their work, Lee S.Y et al. present a comprehensive analysis of the evaluation and therapy of thyroid diseases in pregnant women and the period after childbirth, which includes the management of thyroid cancer [15]. The retrospective study conducted by Yuan X et al. [16] assesses the pregnancy outcomes and neonatal thyroid function in women diagnosed with thyroid cancer. This study offers valuable information regarding the influence of maternal thyroid cancer on both pregnancy and the health of newborns. Mossa M et al. analyze the prognosis of differentiated thyroid carcinoma in pregnant women. This study offers valuable information regarding the prediction and treatment of thyroid cancer in pregnant women [18]. This study conducted a systematic review and meta analysis, led by Korevaar TIM et al. [19], to investigate the relationship between maternal thyroid illness and preterm birth.

This study presents empirical data regarding the influence of maternal thyroid illness on the results of pregnancy and Investigating how thyroid cancer and pregnancy are connected at the molecular level. This study aims to investigate the impact of pregnant hormones and immunological alterations on the progression of thyroid cancer, as well as the reciprocal effect of thyroid cancer on these factors [6]. Developing therapy options to reduce negative effects on both the mother and fetus. This may involve assessing the safety and effectiveness of various treatment methods, including surgery, radioactive iodine therapy, and thyroid hormone replacement therapy, while a woman is pregnant [17]. Investigating fertility preservation choices for women receiving treatment for thyroid cancer, including examining the practicality and results of oocyte or embryo cryopreservation. This could enhance the quality of reproductive counselling and facilitate informed decision-making for women in their reproductive years who have been diagnosed with thyroid cancer [19]. Assessing the enduring impacts of thyroid cancer treatment on the health of both mothers and children. This may entail conducting longitudinal studies to evaluate outcomes such as the rates of recurrence, fertility, complications during pregnancy, and the development of offspring into adulthood. Examining genetic and epigenetic elements that could make individuals more susceptible to thyroid cancer when pregnant or affect how they respond to therapy. Gaining insight into the fundamental genetic pathways may pave the way for tailored methods of diagnosing and treating individuals.

## Conclusions

The case series showcases the clinical situations and treatment strategies observed in pregnant women with a prior history of thyroid cancer. Although it is challenging to balance the medical demands of mothers with cancer and the well-being of their unborn babies, these cases show that successful pregnancies and positive outcomes for newborns can be achieved through a multidisciplinary approach to care. This case series shows the emphasis that, after a cancer diagnosis with appropriate care, childbearing is still possible with regular follow-up.

It also explains the importance of vigilance for growth parameters in the future, appropriate thyroxine supplementation for brain development and follow-up of the thyroid levels (TSH) in the neonate, for follow up. The instances highlight the significance of personalized management regimens customized to the specific medical history, tumor features, and obstetric factors of each patient. In order to further our knowledge of thyroid cancer during pregnancy, it is necessary to do further study. This research should focus on improving our understanding of the condition, developing more effective treatment methods, and minimizing any potential long-term impacts on the health of both the mother and child. By fostering collaboration among oncologists, endocrinologists, obstetricians, and other healthcare professionals, we may work towards enhancing outcomes and the overall quality of life for pregnant individuals diagnosed with thyroid cancer and their children.
